# S-Palmitoylation of Synaptic Proteins as a Novel Mechanism Underlying Sex-Dependent Differences in Neuronal Plasticity

**DOI:** 10.3390/ijms22126253

**Published:** 2021-06-10

**Authors:** Monika Zaręba-Kozioł, Anna Bartkowiak-Kaczmarek, Matylda Roszkowska, Krystian Bijata, Izabela Figiel, Anup Kumar Halder, Paulina Kamińska, Franziska E. Müller, Subhadip Basu, Weiqi Zhang, Evgeni Ponimaskin, Jakub Włodarczyk

**Affiliations:** 1 Laboratory of Cell Biophysics, Nencki Institute of Experimental Biology, Polish Academy of Science, Pasteur Str. 3, 02-093 Warsaw, Poland; a.bartkowiak@nencki.edu.pl (A.B.-K.); m.babraj@nencki.edu.pl (M.R.); k.bijata@nencki.edu.pl (K.B.); i.figiel@nencki.edu.pl (I.F.); paulakaminska97@gmail.com (P.K.); 2 Faculty of Chemistry, University of Warsaw, Pasteura 1, 02-093 Warsaw, Poland; 3 Department of Computer Science and Engineering, Jadvapur University, Kolkata 700032, India; anup21.halder@gmail.com (A.K.H.); subhadip.basu@jadavpuruniversity.in (S.B.); 4 Cellular Neurophysiology, Hannover Medical School, Carl-Neuberg Str. 1, 30625 Hannover, Germany; mueller.franziska@mh-hannover.de (F.E.M.); ponimaskin.evgeni@mh-hannover.de (E.P.); 5 Department of Mental Health, University of Münster, Albert-Schweitzer-Campus 1/A9, 48149 Munster, Germany; wzhang@uni-muenster.de

**Keywords:** posttranslational modifications, palmitoylation, sexes, proteomics, synapses, synaptic plasticity, DHHC7

## Abstract

Although sex differences in the brain are prevalent, the knowledge about mechanisms underlying sex-related effects on normal and pathological brain functioning is rather poor. It is known that female and male brains differ in size and connectivity. Moreover, those differences are related to neuronal morphology, synaptic plasticity, and molecular signaling pathways. Among different processes assuring proper synapse functions are posttranslational modifications, and among them, S-palmitoylation (S-PALM) emerges as a crucial mechanism regulating synaptic integrity. Protein S-PALM is governed by a family of palmitoyl acyltransferases, also known as DHHC proteins. Here we focused on the sex-related functional importance of DHHC7 acyltransferase because of its S-PALM action over different synaptic proteins as well as sex steroid receptors. Using the mass spectrometry-based PANIMoni method, we identified sex-dependent differences in the S-PALM of synaptic proteins potentially involved in the regulation of membrane excitability and synaptic transmission as well as in the signaling of proteins involved in the structural plasticity of dendritic spines. To determine a mechanistic source for obtained sex-dependent changes in protein S-PALM, we analyzed synaptoneurosomes isolated from DHHC7-/- (DHHC7KO) female and male mice. Our data showed sex-dependent action of DHHC7 acyltransferase. Furthermore, we revealed that different S-PALM proteins control the same biological processes in male and female synapses.

## 1. Introduction

Synaptic plasticity plays a fundamental role in the brain since it is essential for learning and memory. Changes in synapse strength are expressed at the level of different synaptic proteins (i.e., receptors, cytoskeleton elements, signaling molecules) and translated into structural and functional modifications of neuronal functions. Many multiple and coordinated signaling pathways that control memory formation at the molecular level have been identified. However, the substantial knowledge of how plastic changes of neurons govern the information processing in the brain comes from the research conducted mainly on the male population [1,2]. Sex should be considered as an important biological variable in neuroscience since sex-dependent differences in the brain are prevalent and can be detected even at the level of single synaptic connections. Divergent synapse molecular organization [3] and signaling pathways [4,5] together with sex-specific changes in plasticity [6,7] may contribute to sex differences in neuronal function and account for sex-related differences in learning and memory [8], emotional responses [9], fear and anxiety. Biological sex has serious clinical consequences and manifests in the existence of gender bias in neuropsychiatric disorders [10]. Females are more vulnerable to develop major depressive disorder [11], while males are at higher risk to suffer from autism spectrum disorder [12]. Moreover, the same disorders exhibit sex-differences in symptoms of severity [13,14]. Despite the obvious importance, current knowledge regarding sex-specific neuroplasticity is poor and mostly focused on the role of estrogen [7,15], calcium/calmodulin-dependent protein kinase [16] in the structural and functional plasticity of dendritic spines and nitric oxide synthase (NOS) [17] impact on synaptic potentiation in both sexes. It is possible that other subtle processes controlling function and activity of synaptic proteins exist and may influence downstream signaling at the synapses, switching alternative signal transduction pathways in females and males. Identification of intracellular mechanisms regulating information processing and storage by neurons in both sexes is critical for the development of sex-specific therapies addressing numerous memory disorders and psychiatric conditions.

Posttranslational modifications (PTMs) are known to be critically involved in assuring proper synapse function. Among multiple PTMs, S-palmitoylation (S-PALM) emerges as a crucial mechanism underlying synaptic integrity and its dysfunction relates to neuropsychiatric disorders [18]. This reversible modification modulates properties of target proteins including neurotransmitter receptors, synaptic scaffolding proteins and secreted signaling molecules what allows quick and precise regulation of synaptic plasticity [19,20,21,22]. One of the palmitoylating enzymes, palmitoyl acyltransferase DHHC7, is of particular interest in relation to the sex-dependent neuroplasticity because of its S-PALM-mediating actions mainly by sex steroid receptors [23]. Functional consequences arising from palmitoylation of estrogen (ER) and progesterone (PR) receptors are expressed in sex-specific changes of synaptic function, plasticity and connectivity of different brain regions [24,25]. Furthermore, DHHC7 is also engaged in the modification of various synaptic proteins [26,27,28] thus regulating their membrane attachment, sorting, and function relevant for the proper function of synaptic connections. However, the distinct and sex-specific mechanism of DHHC7 action is currently unknown. 

Since S-PALM affects a wide range of synaptic proteins some of them could be implicated in sex-specific neuroplasticity, the present study investigated the differences in S-PALM of synaptic proteins between sexes. We have applied a high-throughput proteomic approach—Acyl Biotin Exchange (ABE) method which not only allows pinpointing the S-palmitoylated proteins but also precise sites of S-PALM modifications in those proteins. Using synaptoneurosomal fraction from whole-brain, we showed different S-PALM profiles in female and male wild-type mice (WT). We identified 2458 peptides assigned to 1239 proteins and among all identified S-PALM synaptoneurosomal proteins, 200 were present only in female brains and 271 were identified exclusively in male mice brains. Furthermore, to determine a mechanistic source for sex-dependent changes in S-PALM pattern and examine S-PALM role as a crucial intracellular mechanism governing sex-specific variances in synapse structure and function, we studied S-PALM of synaptic proteins in DHHC-7 knock-out mice of both sexes. We strictly defined synaptic targets for DHHC7 proteins in female and male brains. Our data showed the sex-dependent action of DHHC7 acyltransferase. We identified a total of 150 uniquely S-PALM by DHHC7 proteins in female brains compared with 125 exclusively S-PALM proteins in male brains. Finally, to gain better insight into the biological and functional relevance of the discovered S-PALM synaptic targets, we incorporated bioinformatics analysis. We demonstrated that S-PALM proteins modulate crucial processes for neuronal functioning. Interestingly, the same signaling pathways/biological processes appeared to be regulated by different S-PALM proteins in both sexes.

## 2. Results

### 2.1. Sex-Dependent Differences in S-Palmitoylation of Synaptic Proteins

**Figure 1:**
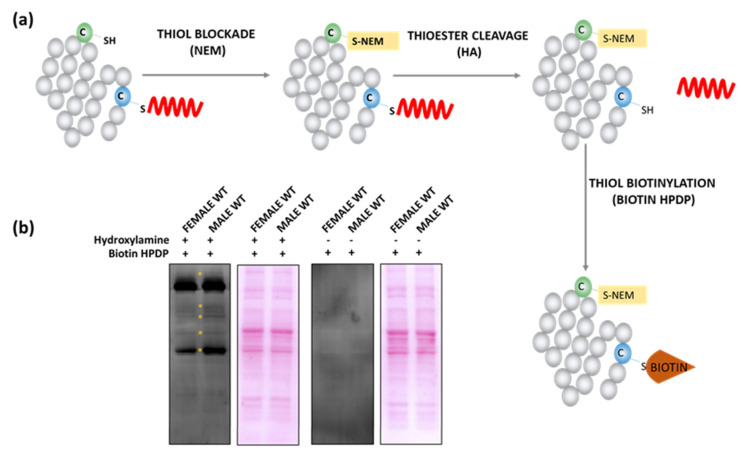
Analysis of S-PALM pattern in the female and male WT synaptoneurosomes. (**a**) Scheme of the applied ABE method. (**b**) Western blot analysis of the pattern of biotinylated proteins obtained with streptavidin-HRP antibody. Controls were prepared without selective cleavage of S-PALM thioester bonds by HA. Ponceau S staining was used as a loading control.

Several studies demonstrate differences between female and male brains at the level of molecular and structural synaptic plasticity, but the underlying mechanisms are not fully understood [1,29]. Looking for the possible source of these differences, we analyzed the S-PALM profile of synaptoneurosomal proteins isolated from three-month-old C57BL/6J littermates, male and female wild type mice (WT). First, we applied the Acyl-Biotin Exchange (ABE) method to check the sex-dependent pattern of synaptic proteins S-PALM. This method is based on the selective cleavage of thioester bonds between cysteines and palmitate by hydroxylamine (NH_2_OH/HA) after blocking free-SH groups by N-ethylmaleimide (NEM) (Figure 1a). Cleavage of the ester linkage allows the specific incorporation of biotin (biotin HPDP) to the newly available thiol group and enables detection by Western blotting. We used non-deacylated samples as controls for the specificity of the reaction. The ABE experiments revealed slight differences in the S-PALM pattern between the sexes (Figure 1b).

**Figure 2:**
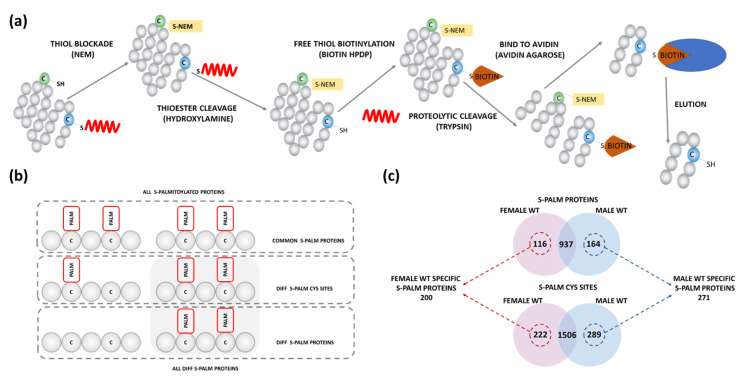
Sex-dependent analysis of protein S-PALM in synaptoneurosomes isolated from female and male WT mice. (**a**) Scheme of PANIMoni mass spectrometry-based method. (**b**) Scheme of S-PALM analysis showing differential and sequential S-PALM classification. (**c**) Venn diagram analysis of differential S-PALM in female and male synaptoneurosomes (Nmice = 3/group).

To identify differentially S-PALM proteins in both sexes, we used the mass spectrometry-based approach developed by us earlier, the PANIMoni method (see Materials and Methods for details) [30]. Similarly to the ABE, S-PALM proteins are labeled with biotin. This is followed by proteolytic digestion prior to the capture by avidin. This step allows the selective isolation of previously S-PALM peptides but does not detect intact S-PALM proteins. The PANIMoni method allows finding changes in S-PALM at the level of specific proteins and cysteine residues (Figure 2a). A standard control that omits the HA-driven cleavage of acyl bonds allows the separation of false identifications.

Using this method combined with peptide identification by mass spectrometry, we recognized 2017 peptides in the synaptoneurosomal fraction that are assigned to 1217 S-PALM proteins with less than 1% false discovery rate (FDR) (Table S1). Besides, the high similarity of S-PALM identification was confirmed in three biological replicates (Nmice = 3/per group) by mass spectrometry. The overall scheme of differential S-PALM analysis and classification of synaptoneurosomal proteins into different sets of proteins is presented in Figure 2b.

Among all identified S-PALM synaptoneurosomal proteins, 116 were present exclusively in female brains, while 164 were identified only in male mouse brains. We also observed differences at the level of specific sites of S-PALM. Altogether, we identified 200 S-PALM synaptic proteins upregulated in female synaptoneurosomes and 271 S-PALM synaptic proteins that were upregulated in male derived synaptoneurosomes (Figure 2c).

**Figure 3:**
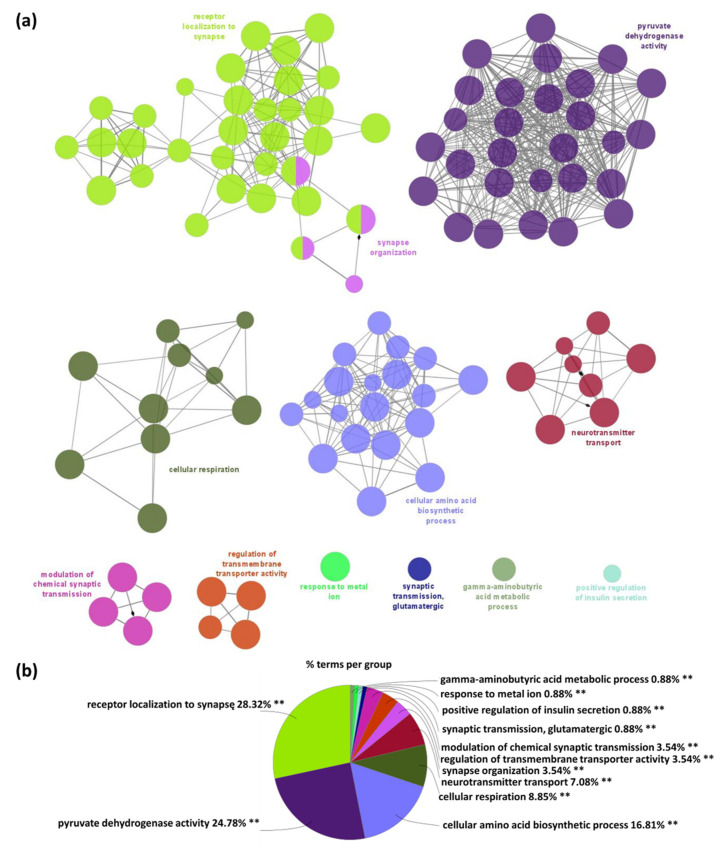
Functional enrichment analysis of proteins specific for female WT mice using the Gene Ontology biological processes database and the Clue Go algorithm. (**a**) Network depicting interactions between enriched functional classes. Each circle represents a biological term consisting of various related proteins/genes. Terms that belong to the same pathway are shown with the same color, and terms associated with two different pathways are marked with two colors. The size of the circles relates to the statistical significance of the term enrichment. The connectivity (edges) between the terms in the network is derived from kappa score, (indicates the similarity of associated genes shared by different terms). Thicker edges indicate stronger similarity. Diamonds represent directed edges which link parent terms to child terms. Only the name of the most significant term in each group is shown to reduce overlay. (**b**) A diagram showing the percentage of terms per group of enriched protein classes.

To gain functional insight into the S-PALM-proteins which differentiate between female and male WT mice, we applied the ClueGO, the widely used Cytoscape plugin [31,32]. In WT female synaptoneurosomes, 113 Gene Ontology biological processes (GO_BP) terms were significantly enriched (*p*-value < 0.01) among WT female specific proteins and categorized into 11 GO groups (networks) as shown in Figure 3a. The most presented GO_BP significant functional groups included: receptor localization to synapse (*p*-value = 0.00001, e.g., Dlg4, Git1, Gphn), synapse organization (*p*-value = 2.01 × 10^−7^, e.g., Dlg4, Nectin1, Septin7, Shank3), pyruvate dehydrogenase activity (*p*-value = 5.34 × 10^−8^, e.g., Dld, Pdha1, Pdk3), cellular respiration (*p*-value = 3.85 × 10^−8^, e.g., Aco2, Ndufb9, Ndufv1), neurotransmitter transport (*p*-value = 7.77 × 10^−7^, e.g., Nrxn1, Septin5, Slc25a22), regulation of transmembrane transporter activity (*p*-value = 0.00002, e.g., Gja1, Homer1, Park7, Tcaf1), and other (Figure 3b). All GO_BP terms (*p* < 0.01) along with the percentage of genes associated with upregulated S-PALM synaptic proteins in the female WT are presented in Figure S1.

**Figure 4:**
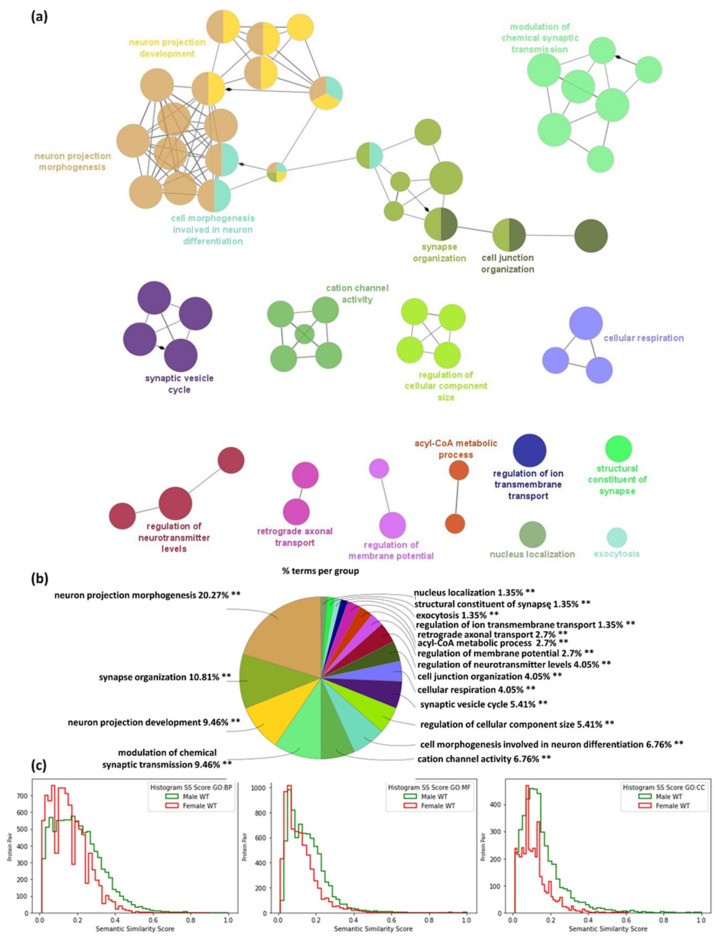
Functional enrichment analysis of proteins specific for male WT mice using the Gene Ontology biological processes database and the Clue Go algorithm. (**a**) Network depicting interactions between enriched functional classes. (**b**) A diagram showing the percentage of terms per group analysis of enriched protein classes. (**c**) Comparison of semantic similarity (SS) of protein pairs from male and female WT mice for each type of Gene Ontology term: cellular components (CC), molecular functions (MF) and biological processes (BP), respectively. The *X*-axis represents the SS score (ranges—0, 1) and the *Y*-axis represents the frequency of protein pairs.

Similar analysis of specific S-PALM synaptic proteins in WT male mice is presented in Figure 4. In contrast to females, in male synaptoneurosomes 74 GO_BP functional groups were significantly enriched (*p* < 0.01) and grouped into 17 GO terms networks, as presented in Figure 4a and Figure S2.

The most significant GO_BP pathways enriched (*p*-value < 0.01) in the 155 male-specific S-PALM proteins set were related to: neuron projection morphogenesis (*p*-value = 2.12 × 10^−7^, e.g., Bcan, Cdc42, Flot1, Nptn), neuron projection development (*p*-value = 1.51 × 10^−9^, e.g., Camk1, Camk2g, Nptn), modulation of chemical synapse transmission (*p*-value = 4.07 × 10^−6^, e.g., Gria4, Grik2, Grin2b, Snap25), but also synaptic vesicle cycle (*p*-value = 1.71 × 10^−8^, e.g., Prkaca, Rap1b, Rims1, Slc32a1, Snap25), synapse organization (*p*-value = 0.0058, e.g., Camk1, Cdc42, Grin2b, Srcin1), and others (Figure 4a,b).

Additionally, we quantified pairwise similarities between protein annotations based on semantic similarity measure for Gene Ontology terms proposed by Dutta et al. [33]. The ontological annotations of each protein pair were incorporated into a graph-theoretic approach for assessing the SS score. Our analysis shows that the distribution of semantic similarities between protein pairs of the male WT are not from the same distribution as the female WT (Figure 4c).

**Figure 5:**
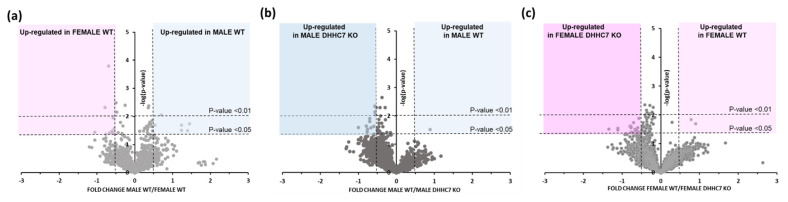
Sex-dependent differences in synaptic protein expression. Volcano plots display differentially regulated synaptic proteins of female and male WT mice (**a**), male WT and male DHHC7 KO (**b**), and female WT and female DHHC7 KO (**c**). Proteins with statistically significant differential expression (*p* < 0.05) are highlighted in the top right and left quadrants.

To verify that the observed changes were not due to a difference in protein expression, we compared the expression levels of synaptoneurosomal proteins from the brains of female and male WT mice. Our analyses showed slight changes in protein expression between the compared groups (Table S2). Overall, in the proteomics experiments, we identified a total of 6250 peptides which are assigned to 2592 proteins with FDR 1%. Among 2592 identified and quantified synaptoneurosomal proteins, a minority of them showed differential expression, with 17 proteins found to be significantly upregulated in female WT (e.g., Slc6a1, Sept10, Asap2,) and 8 in male WT (e.g., Reep2, Skp1a or Crat) (Figure 5a, Table S2).

### 2.2. DHHC7–Dependent Synaptic Proteins S-Palmitoylation in Male and Female Mice

To better understand the mechanisms underlying the sex-dependent S-PALM, we used DHHC7 KO [34,35]. DHHC7 is not only involved in S-PALM of different synaptic substrates but is also responsible for modifying sex steroid receptors [36,37]. Moreover, it was shown to be developmentally regulated in a sex-dependent manner [23]. To determine a mechanistic source for the sex-dependent activity of DHHC7, we examined S-PALM of synaptic proteins in female and male DHHC7 KO mice. 

In the first step, we found that the vast majority of proteins did not show significant changes in expression between WT and DHHC7 KO synaptoneurosomal brain tissues in females and males (Figure 5b,c). However, differential analysis revealed that a small handful of proteins exhibit differential expression, with one protein (Rmnd5b) which was significantly increased in male DHHC7 KO. In contrast, 15 proteins (e.g., Prkcd, Slc18a2) were found to be significantly increased in male WT synaptoneurosomes (Figure 5b and Table S2). Similarly, when we compared the synaptic proteins of female WT and DHHC7 KO, we noticed 17 proteins upregulated in WT female mice (e.g., Kcnma1) and 6 proteins upregulated in female DHHC7 KO (e.g., Atp1a1, Dld, uba1a) (Figure 5c and Table S2).

**Figure 6:**
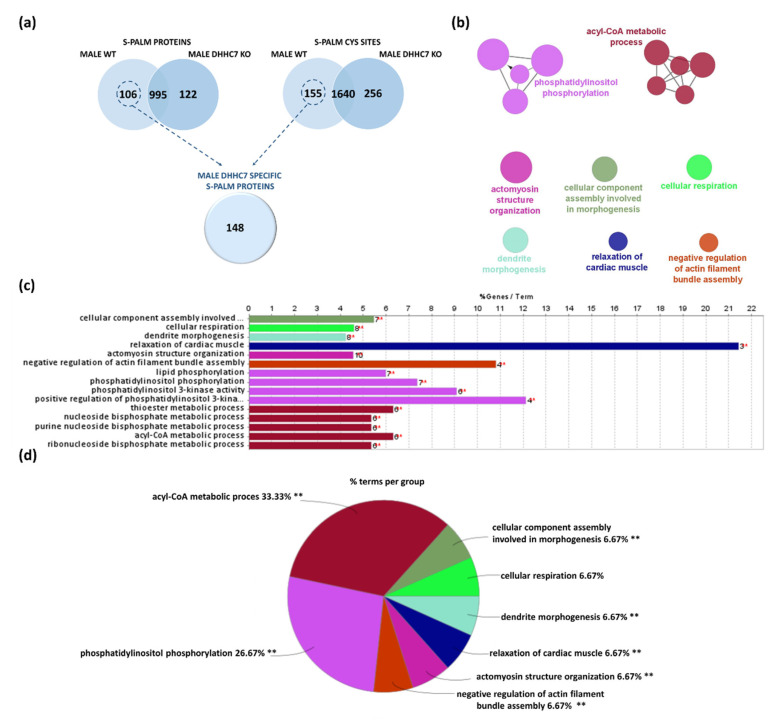
Analysis of male synaptic proteins regulated by DHHC7 using the Gene Ontology biological processes database and the Clue Go algorithm. (**a**) Venn diagram analysis of S-PALM in male WT and male DHHC7 KO synaptoneurosomes. (**b**) Network depicting interactions between enriched functional classes. (**c**) List of all enriched GO_BP functional classes (*p* < 0.01) showed on the network. (**d**) Percentage of terms per group analysis of enriched protein classes.

To assess the role of DHHC7 in the palmitoylation of synaptic proteins, we applied the PANIMoni proteomic method to synaptoneurosomes isolated from DHHC7 KO male brains (Figure 6a, Table S3). In total, we identified 1896 peptides that correspond to 1117 proteins. To find the degree of similarity between the identified protein groups (male WT and male DHHC7 KO) we used Venn diagram analysis. Proteins relying on DHHC7 for their palmitoylation were expected to be absent from the palmitoyl proteomes of DHHC7 KO. Among all identified S-PALM synaptosomal proteins, 106 proteins were present only in male WT, while 122 proteins were found only in male DHHC7 KO (Figure 6a). Additionally, we detected the differences at the level of specific S-PALM sites. Overall, combining the protein and site identification data, we indicated 148 proteins with S-PALM regulated by DHHC7 in male synaptoneurosomes (Figure 6a). Proteins identified only in male DHHC7 KO may represent proteins that are modified by some undefined compensation mechanisms.

In order to decipher the molecular mechanisms at the synapse in which DHHC7-dependent S-PALM in males might play a distinct role, we performed functional enrichment analyses of terms from the GO_BP using the ClueGO algorithm. Analyses of this set of protein comprising 148 proteins revealed multiple enriched functional categories (Figure 6b,c). Terms related to: phosphatidylinositol phosphorylation (*p*-value = 0.00034, e.g., Cdc42, Erbb4, Ptk2b), acyl-CoA metabolic process (*p*-value = 0.0039, e.g., Acot1, Dld, Pdha1, Suclg1), actomyosin structure organization (*p*-value = 0.00024, e.g., Cdc42, Csrp1, Epb41l1, Pdgfra, Zyx), cellular component assembly involved in morphogenesis (*p*-value = 0.00003, e.g., Ckap5, Clasp2, Csrp1, Nfasc, Ttn), but also cellular respiration (*p*-value = 0.0022, e.g., Dld, Mtch2, Ndufs1, Ndufv1), dendrite morphogenesis (*p*-value = 0.0041, e.g., Cdc42, Cdkl3, Shank1), relaxation of cardiac muscle (*p*-value = 0.0082, e.g., Atp2a2, Camk2g, Ttn), and negative regulation of actin filament bundle assembly (*p*-value = 0.0091, e.g., Clasp2, Coro2b, Dbn1, Shank1) were significantly (*p*-value < 0.01) statistically enriched (Figure 6c).

**Figure 7:**
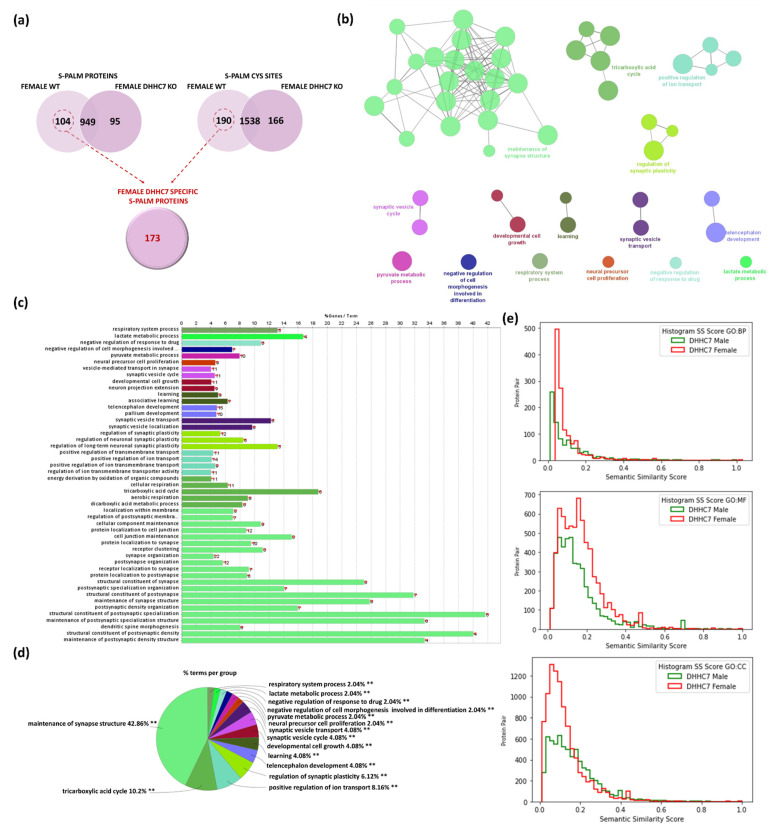
Analysis of female synaptic proteins regulated by DHHC7 using the Gene Ontology biological processes database and the Clue Go algorithm. (**a**) Venn diagram analysis of S-PALM in WT and DHHC7 KO female synaptoneurosomes. (**b**) Network depicting interactions between enriched functional classes. (**c**) List of all enriched GO_BP functional classes (*p* < 0.01). (**d**) Percentage of terms per group analysis of enriched protein classes. (**e**) Comparison of semantic similarity (SS) of protein pairs from DHHC7 male and female-specific proteins for each type of Gene ontology terms, cellular components (CC), molecular functions (MF) and biological processes (BP) respectively. *X*-axis represents the SS score (ranges [0, 1]) and the *Y*-axis represents the frequency of protein pairs.

Similarly, we analyzed S-PALM in synaptoneurosomes isolated from DHHC7 KO female brains. A total of 1894 distinct peptides assigned to 1149 S-PALM proteins were identified and quantified with less than 1%FDR (Table S4). The S-PALM profiles of female DHHC7 KO and female WT synaptoneurosomes were comparatively analyzed to identified differentially modified proteins (Figure 7a). Combining the protein and site identification data, we revealed that 173 proteins from female synaptoneurosomes are dependent on DHHC7 for their palmitoylation. Additionally, in the case of female synaptoneurosomal proteins, we observed that the lack of DHHC7 leads to an increased number of S-PALM proteins in synaptoneurosomes isolated from DHHC7 KO. To classify proteins regulated by DHHC7 in female brains, we analyzed a set of previously distinguished 173 synaptic proteins, again using the Gene Ontology and ClueGO algorithm (Figure 7a). Our analysis revealed that the proteins modulated by DHHC7 in female mice belong to some different functional categories Figure 7b,c. Importantly, several well-defined categories related to brain plasticity were significantly enriched, such as: maintenance synapse structures (*p*-value = 2.38 × 10^−8^, Bsn, Dlg2, Dlg4, Syngap1), tricarboxylic acid cycle (*p*-value = 0.00004, e.g., Dlat, Idh2, Ogdh, Pdha1), positive regulation of ion transport (*p*-value = 0.0002, e.g., Atp2b2, Cacna2d1, Dpysl2, Kcna1), regulation of synaptic plasticity (*p*-value = 0.00008, e.g., Dlg4, Grik2, Kras, Shank1), synaptic vesicle transport (*p*-value = 0.0005, e.g., Dnm1, Dnm3, Dpysl2, Rab3a), and other (Figure 7b,c).

Additionally, we used the semantic similarity score to measure the redundancy of the identified terms within each dataset. The histogram distribution plots for the SS scores for all three GO terms graphs for male and female DHHC7 specific proteins are presented in Figure 7e.

In conclusion, we revealed the substrate specificity of the DHHC7 protein using the PANIMoni mass spectrometry-based approach in female and male mice synapses.

### 2.3. DHHC7 Operates Differently in Male and Female Mice

**Figure 8:**
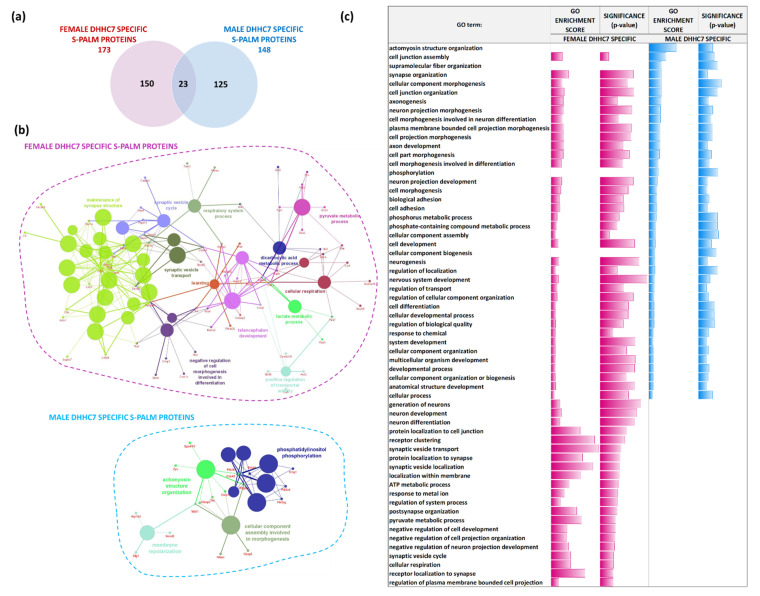
Analysis of female synaptic proteins regulated by DHHC7 using the Gene Ontology biological processes database and the Clue Go algorithm. (**a**) Venn diagram analysis of S-PALM in female WT and male DHHC7 KO synaptoneurosomes. (**b**) Networks depicting interactions between enriched functional classes for male and female DHHC7 specific proteins. (**c**) Summary of all enriched GO_BP functional classes for male and female DHHC7 specific proteins. (*p* < 0.05) with depicted fold change and significance of enrichment.

One of the main topics of the present study was to resolve sex-specific differences in the synaptic S-palmitoylome. Thus, we compared the sets of proteins identified as regulated exclusively by DHHC7 in male (148 proteins) and female (173 proteins) synaptoneurosomes. Interestingly, we observed slight overlap of DHHC7-dependent synaptic proteins between males and females. We found that only 23 S-PALM proteins were commonly regulated by DHHC7 in female and male synaptoneurosomes, while 125 were exclusively modified in males and 150 in females (Figure 8a).

We then analyzed which pathways are regulated by the identified DHHC7 dependent S-PALM proteins in male and female synaptoneurosomes (Figure 8b). Regarding proteins regulated by DHHC7 in females, the GO analysis of biological processes showed that proteins involved in maintenance synapse structures (*p*-value = 2.812 × 10^−7^, e.g., Bsn, Dlg2, Dlg4, Dlgap1, Gphn, Rab3a, Syngap1), cellular respiration (*p*-value = 0.0006, e.g., Cyct, Dlat, Hif1a, Idh2, Ndufb9, Ogdh, Park7, Sdha, Slc25a18), and synaptic vesicle cycle and transport (*p*-value = 0.00138, e.g., Bsn, Cadps2, Ctnnb1, Dnajc6, Dnm1, Dnm3, Napb, Ptpn11, Rab3a, Rap1a) are the most enriched at the synapse.

Contrary to females, in males the most enriched proteins dependent on DHHC7 are proteins involved in processes related to: actomyosin structure organization (*p*-value = 0.00022, e.g., Cdc42, Clasp2, Csrp1, Epb41l1, Pdgfra, Ptk2b, Ttn, Wdr1, Zyx), membrane repolarization (*p*-value = 0.0037, e.g., Atp1b3, Dlg1, Kcnd3, Wdr1), and other.

Next, we summarized all of the enriched terms of GO_BP (*p*-value < 0.05) from each set. Interestingly, we found that these sex-specific sets of proteins are involved in the regulation of the same biological processes but differ in their enrichment levels (Figure 8c). In conclusion, we demonstrated that the common signaling pathways of both sexes appear to be regulated by different S-PALM proteins.

## 3. Discussion

Research over the last few decades has provided evidence that sex differences are more widespread than previously supposed. The interest in the differences between males and females concerns not only brain morphology and neurocognitive functions, but also epidemiology and clinical expression of the neurological and psychiatric disorders [2,8,12,13,38,39]. In our work, we used sophisticated proteomics and bioinformatics tools to study sex-dependent differences in biological processes related to protein S-PALM in synaptoneurosomes. We focused our research on the sex-dependent functions of one of the enzymes governing the S-PALM, the palmitoyl acyltransferase DHHC7. Despite the exceptional sensitivity in identifying S-PALM modification sites in proteins, MS-based indirect approaches have some limitations. Rigorously reproducible sample preparation is the basis of all differential proteomic studies and is especially important in the differential analysis of unstable PTMs. It is well established that the protein S-PALM analyzed in this work possesses unique reactivity. It is also known that transpalmitoylation and depalmitoylation reactions, which lead to artefacts in the identification of S-PALM proteins may occur in the presence of an activated fatty acyl thioester so that the various substrate cysteine nucleophiles can attack. False-positive results are often observed but rarely reported due to deletion at the level of data analysis. To eliminate false positives, we performed a negative control and implemented very stringent analysis criteria for selection of S-PALM proteins. It is well-known that S-PALM modulates the functions of synaptic proteins involved in neuronal development, plasticity, and also those related to synaptic dysfunction, thus leading to neurological diseases [18,20,35,40,41]. Our results showed, for the first time, that synaptic proteins are differentially regulated by S-PALM in male and female synapses, which may be the source of sex differences in signaling pathways in the brain. Moreover, we demonstrated that DHHC7 acyltransferase acts in a sex-dependent manner and modulates different proteins in the brains of female and male mice. We showed that 150 proteins were exclusively S-palmitoylated by DHHC7 in females, while 125 in male synapses. Our bioinformatics analysis clearly showed that S-PALM-dependent signaling pathways found in both sexes are modulated by different S-PALM proteins. At the same time, we observed slight changes in protein expression between sexes. However, these changes did not affect S-PALM results.

Sex-dependent differences have been described in the neuronal structure, dendritic branching, as well as in the morphology and density of dendritic spines [2,29,42]. Sex-related changes have been also investigated at the level of brain proteome [43,44,45,46]. Distler et al. reported a profile of sex-specific synaptic proteins for different regions of the adult mouse brain, namely the hippocampus, cerebellum, prefrontal cortex, and striatum [43]. It was also shown that the intersexual alterations in hippocampal protein expression pattern are related to those observed in behavioral tests [44]. Moreover, the differences in the proteomic profiling of the mouse hippocampus were shown to depend not only on the sex but also on the age of animals [45].

PTMs that modulate protein function are also involved in the control and regulation of synaptic processes [47,48,49]. One important aspect of the research on brain sexual dimorphism was finding the changes in S-nitrosylation (S-NO) of mouse cortical proteins. Mass spectrometry analysis revealed that female mice showed elevated levels of S-NO proteins involved in synaptic processes, while males exhibited higher enrichment of the S-NO-dependent cytoskeletal pathways [50]. S-NO is engaged in a variety of cellular processes and, importantly, this PTM appears to compete with S-PALM for cysteine residues in proteins [51,52]. In our previous study, we reported that proteins crucial in proper synapse functioning can undergo atypical crosstalk between the S-PALM and S-NO, which can result in the development of chronic stress disorder [30]. Hence, both aforementioned PTMs of synaptic proteins appeared to be an important mechanism for controlling normal brain function in both sexes.

Although it was reported that DHHC7 modulates S-PALM of several synaptic proteins, its role in the regulation of synaptic plasticity is still elusive. Recently, DHHC7 deficiency has been shown to impair excitatory transmission, synaptic plasticity, and hippocampal structural connectivity [34]. Additionally, the authors reported sex-related differences in the hippocampal microstructure as well as in synaptic transmission in the medial prefrontal cortex of DHHC7 KO mice [34]. Here, using the same mouse model, we showed that the observed changes might arise from divergent substrate specificity of DHHC7 acyltransferase in male and female synapses. We found that proteins specifically regulated by DHHC7 in females show significantly higher enrichment in processes such as synapse structure maintenance, synaptic vesicle cycle and transport, cellular respiration, or learning.

The molecular basis of sex-dependent changes in neuronal function remains elusive. Several data have shown that sex-differences in synaptic plasticity are regulated by both direct and indirect mechanisms of steroid hormone action [53]. A growing body of evidence shows that sex steroids can alter these processes [54,55,56,57].

It has been repeatedly shown that estrogen enhances synaptogenesis and modulates synaptic transmission [55,57,58]. Alterations in spine density in response to sex steroid hormone fluctuations across the estrous cycle in female rodents have been observed [59,60,61]. Female neurons in general are characterized by a higher dendritic spine density in comparison to males, suggesting that the sex difference may be due to the effects of sex steroids [62,63]. Sex-specific changes in spine density were observed in different brain regions, such as the hippocampus, nucleus accumbens, or the prefrontal cortex [63,64]. Moreover, it was shown that estradiol can potentiate excitatory synapses and attenuate inhibitory synapses exclusively in females [58,59,65,66,67]. In contrast, testosterone appears to inhibit long term potentiation (LTP) and dendritic sprouting in the male hippocampus [68]. Taken together, all these data provide evidence that sex steroid hormones regulate synaptic plasticity in a sex-specific manner. Here, we postulate that DHHC7-mediated palmitoylation of synaptic proteins underlies some of the sex-related differences in neuronal plasticity. Recent studies have shown that S-PALM is an important regulator of synaptic transmission, synaptic vesicle cycle and activity of synaptic receptors [18,69,70]. In our study, we identified a group of proteins palmitoylated by DHHC7 acyltransferase that are upregulated in female synapses. This group includes proteins associated with the maintenance of synapse structure, such as gephyrin, Rab3a, and Syngap1. It is widely known that the S-PALM of these proteins dynamically regulates interactions with other proteins, and thus participates in synaptic stability and trafficking [71,72,73].

Gephyrin is an essential scaffolding protein that forms post-synaptic clusters at inhibitory synapses [74]. S-PALM has been found to be critical for both the stable aggregation of gephyrin and inhibitory synaptic transmission [71]. A recent study demonstrated that S-PALM of gephyrin potentiated GABAergic synaptic transmission by increased amplitude of miniature inhibitory postsynaptic currents. Moreover, it was shown that DHHC7 KO female mice exhibited increased inhibitory transmission while male DHHC7 KO displayed reduced inhibitory transmission [34,71]. Our study suggests that these sex-specific effects may be regulated by S-PALM of gephyrin.

Ras-related protein Rab-3A (Rab3a) is a small GTP-binding protein that plays a crucial role in exocytosis and regulation of secretion. It was reported that the expression of Rab3a protein in the rat pituitary gland is regulated by long term estrogen therapy [75]. However, the effects of Rab3a S-PALM have never been studied. Therefore, our findings could be the starting point of the research into the palmitoylation role in the function of this important protein.

Syngap1 is a Ras/Rap GTPase activating protein that is specifically expressed in neurons and highly abundant at glutamatergic synapses in the brain [76]. Interestingly, a decrease in SynGAP1 concentration correlates with changes in the postsynaptic density (PSD) composition exclusively in females [77]. Although S-PALM of this protein was previously reported, the influence of this PTM on protein activity is unknown.

The second highly enriched biological process in female is cellular respiration. Mitochondria exhibit sex-dependent activity at various levels, including oxidative capacities, calcium handling, and resistance to oxidative stress [78]. Accumulating evidence indicates that brain mitochondria are targets for steroids [79]. For example, it was shown that the NADH-related respiratory rate was higher in females than in males [80]. Additionally, a lower level of oxidative stress was found in mitochondria of young females compared with males. Nevertheless, the brain mitochondria of young male mice have a more efficient glutathione cycle than female mice [80]. In contrast, mitochondria from the female brain have higher activity of the electron transport chain, increased ATP production, and higher functional capacities [81]. 

In our study, we confirmed that the succinate dehydrogenase (Sdha) is specifically modified by DHHC7 in female mice. Sdha belongs to the oxidative phosphorylation system in mitochondria and connects the TCA cycle to the electron transport chain. Harish et al. demonstrated that the activity of Sdha protein in the human brain was significantly higher in females compared with males [82]. Here, we also identified a pyruvate dehydrogenase Pdha1 that catalyzes the conversion of pyruvate to acetyl-CoA and CO_2_ and links the glycolytic pathway to the TCA. This protein is much more active in the brains of female mice as compared with those of male [83].

We also found that, unlike female synapses, the synapses of male mice were characterized by significant enrichment of actomyosin structure organization, morphogenesis, and enhanced phosphatidylinositol phosphorylation and membrane repolarization. 

The myosin-actin interaction is essential for regulating cell growth. Actin filaments support the structure of dendritic spines, and changes in actin dynamics are known to mediate synaptic plasticity [84]. Myosin activity is particularly important during synaptogenesis, but little is known about the role of motor proteins in mature synapses and synaptic plasticity. It has been reported that estradiol and progesterone promote actin cytoskeleton remodeling, causing morphological changes in dendritic spines [15,85]. Noteworthily, testosterone is also involved in the regulation of cytoskeletal proteins in the brain [85].

In our study, we identified proteins engaged in actomyosin structure organization, modified specifically in male mice, such as Cdc42, zyxin, and Casp2. The small GTPase Cdc42 is a major regulator of actin cytoskeleton, and thus modulates neuronal morphology [86,87]. A number of studies indicate that S-PALM regulates the activity of Cdc42, promoting dendritic spine stabilization [21,88,89]. It was shown that estrogen receptor β signaling may lead to activation of the Cdc42/Rac-dependent pathway that results in changes in the number of dendritic spines [90]. Additionally, it is known that testosterone triggers the activation of Cdc42 in cancer cells [91].

Another protein we identified was, a non-receptor protein-tyrosine kinase (Ptk2b/Pyk2), which regulates reorganization of the actin cytoskeleton, cell polarization, and cell migration [92]. Ptk2b/Pyk2 modulates hippocampal excitatory synaptic transmission and contributes to cognitive deficits [93]. However, the sex-dependent activity of this protein has never been studied.

We also identified a group of proteins including Atp1b3, Dlg1 (SAP97), and Kcnd3 that govern membrane repolarization and are specifically regulated by DHHC7 in males. SAP97 is a member of MAGUK family of proteins that play a major role in the trafficking and anchoring of potassium channels to the plasma membrane. These channels are essential for maintaining resting membrane potential, repolarizing action potential and mediating cell excitability [94]. Interestingly, S-PALM SAP97 is directed to the PSD, where it regulates the distribution of AMPA receptors and, hence, influences the synaptic strength [95].

Our research shows that, with the exception of highly enriched biological processes that are unique to females or males, there are several biological processes that are common to both sexes but regulated by different proteins. We can conclude that the same signaling pathways/biological processes appeared to be regulated by different palmitoylated proteins in both sexes. This is an interesting observation because, to the best of our knowledge, the role of S-PALM in the sexual dimorphism of synaptic processes in the brain has not been described previously.

Taken together, the presented mass spectrometry-based high throughput analysis revealed significant sex-dependent differences in the protein S-PALM and the biological processes controlled by this PTM. We unraveled unique protein substrates for DHHC7 along with their specific S-PALM sites in a sex-related manner. Moreover, we demonstrated that different S-PALM proteins control the same biological processes in male and female synapses. Our data provide a unique, mechanistic understanding of the role of S-PALM in females and males. The results of this study unravel sexual dimorphism in S-PALM regulation of brain functions, especially in synaptic signaling pathways. 

Furthermore, our findings underline the necessity of including both males and females in all experimental studies. In many cases, sex may influence the major outcome of the research. 

## 4. Materials and Methods

### 4.1. Animals and Ethical Statement

In experiments, we used 90-day-old mice C57BL/6J (females and males) wild type (WT) and DHHC7 KO housed in groups [34,35]. Experiments were approved by the local institutional animal care and research advisory committee and permitted by the Lower Saxony State Office for Consumer Protection and Food Safety (LAVES; file number 16/2230) The study was performed in accordance with all relevant guidelines and regulations of German animal protection law and with the European Directive 2010/63/EU.

### 4.2. Synaptoneurosomes

Synaptoneurosomes were prepared from the brains of WT and DHHC7 KO (males and females Nmice/group = 3) as previously described [30,96]. Briefly, after euthanasia by cervical dislocation, the mice were decapitated. Brains were homogenized with Dounce homogenizer in 3 mL of buffer A (5 mM HEPES (pH 7.4), 0.32 M sucrose, 0.2 mM ethylenediaminetetra acetic acid (EDTA), 50 mM N-ethylmaleimide (NEM), and protease inhibitor cocktail. Nuclei and cell debris were pelleted by 5 min centrifugation at 2500× *g*. Supernatant was then centrifuged at 12000× *g* for 5 min. The obtained pellet fraction was layered over a discontinuous Ficoll (Sigma Aldrich) gradient (4%, 6%, and 13%), and centrifuged at 70,000× *g* for 45 min. The synaptoneurosomal fraction was collected in buffer A and centrifuged at 20,000× *g* for 20 min. The pellet corresponded to the synaptoneurosomes fraction. The purified synaptoneurosomes were used in all experiments.

### 4.3. Acyl-Biotin Exchange (ABE)

To the changes in the S-PALM of proteins, acyl-biotin exchange (ABE) was used. Synaptoneurosomes were dissolved in the buffer that contained 50 mM Tris HCl (pH 7.5), 150 mM NaCl, 1 mM EDTA, 4% SDS and 1% Triton X-100. Next, to block free thiol groups samples were incubated with 50 mM N-ethylmaleimide at 4 °C for 16 h with agitation. The thioester bonds between SH and palmitic acid were decomposed using the selective agent 1 M hydroxylamine, and newly formed thiols were blocked with 400 µM cysteine-specific biotin-HPDP (N-[6-(biotinamido)hexyl]-3′-(2′-pyridyldithio)propionamide). The ABE technique combined with immunoblotting analysis was used for S-PALM pattern analysis.

### 4.4. PANIMoni

PANIMoni analysis was performed as described previously [30]. The biotin labeling of S-PALM proteins in lysates was performed based on ABE procedure. Protein fractions that contained biotinylated proteins were digested using sequencing-grade modified trypsin (Promega V 5111) for 16 h at 37 °C. Digestion was terminated using a protease inhibitor cocktail. The tryptic peptide mixture was incubated with 100 μL of NeutrAvidin beads at room temperature for 1 h. The NeutrAvidin beads were washed five times in 1 mL of wash buffer (50 mM Tris (pH 7.7), 600 mM NaCl 0.2 mM EDTA). Neutravidin-bound peptides were eluted with 150 μL of elution buffer and 5 mM TCEP and concentrated in a SpeedVac. Trifluoroacetic acid was added to the peptide solution to achieve a final concentration of 0.1%. The samples were analyzed by nanoLC-MS and nanoLC-MS/MS.

### 4.5. Mass Spectrometry

The S-PALM or all proteins peptide mixture (20 µL) was applied to the nanoACQUITY UPLC Trapping Column (Waters, 186003514) using water containing 0.1% formic acid as the mobile phase and transferred to the nanoACQUITY UPLC BEH C18 Column (75 µm inner diameter; 250 mm long, Waters 186003545) using an acetonitrile gradient in the presence of 0.1% formic acid with a flow rate of 250 nL/min. The column outlet was directly coupled to the ion source of the Thermo Orbitrap Elite mass spectrometer (Thermo Electron Corp., San Jose, CA, USA) working in the regime of data-dependent MS to MS/MS switch. HCD fragmentation was used.

All MS runs were separated by blank runs to reduce the carry-over of peptides from previous samples. Results of measurements were processed using Mascot-Distiller 2.7.1 software (MatrixScience, London, UK, on-site license). The Mascot search engine (version 2.7.1) was used to survey data against the UniProtKB/Swiss-Prot database (Swissprot 2020_02; 16,905 sequences). The search parameters were set to the following: taxonomy (*Mus musculus*), variable modifications (cysteine carbamidomethylation or N-malemideidation, methionine oxidation, peptide tolerance (5 ppm), fragment mass tolerance (5 ppm). Enzyme specificity: trypsin with one missed or nonspecific cleavages permitted. The mass calibration and data filtering described above were also carried out. The lists of the peptide sequences (SPL) that were identified in all of the LC-MS/MS runs from females (WT and DHHC7 KO) and males (WT and DHHC7 KO) synaptoneurosomal fractions were merged into one peptide list using MascotScan software (http://proteom.ibb.waw.pl/mscan/, accessed on 9 April 2021). The SPL consists of sequences of peptides with Mascot scores exceeding the threshold value corresponding to 5% expectation value and FDR1% calculated by Mascot procedure. For proteome quantitative analysis, peptides intensities were determined as the surface of the isotopic envelope of the tagged isotopic envelopes. Before the analysis, quantitative values were normalized with LOWESS as described previously [30,95].

### 4.6. Functional Bioinformatics Analysis

For integrative analysis, we used the ClueGO software to observe differential proteins involved in the GO terms. The input list of proteins for each GO analysis were distinguished at the basis of proteo-mic data analysis and Venn diagram analysis. The lists of specific proteins are grouped in Tables S1–S4. Proteins were analyzed with ClueGO v2.6.4/CluePedia v1.6.5 to achieve complete Gene Ontological terms (GO) from our datasets [31]. ClueGO integrates GO terms and creates an organized GO/pathway term network. The statistical test used for the nodes enrichment was based on right-sided hypergeometric option with a Benjamini-Hochberg correction and kappa score of 0.5. As a reference set for term enrichment calculations we utilized genes from *Mus musculus* genome (NCBI unique Gene identifiers). Enrichment of GO was conducted for different sets of proteins, and *p*-values < 0.05 were considered to be significant. All ClueGO results are grouped in the Table S5.

### 4.7. Semantic Similarity (SS) Analysis

SS analysis between the protein pairs was performed using three genes ontological (GO) relationship graphs (CC, MF and BP) based on the SS measure proposed by Dutta et al. [33]. The ontological annotations of each protein pair were incorporated into a graph-theoretic approach for assessing the SS score. GO terms were grouped into three independent direct acyclic graphs where nodes represent specific GO terms and the links among nodes represent different hierarchical relationships (‘is_a’, ‘part_of’, and ‘has_part’) between the GO terms. To compute the SS score between two proteins, the semantic similarity was estimated for all the GO term pairs associated with the two proteins. A greater number of similar GO annotations between the two proteins indicates a higher SS score between the proteins.

## Data Availability

Data are available via ProteomeXchange with identifier PXD025286.

## Supplementary Materials

The following are available online at https://www.mdpi.com/article/10.3390/ijms22126253/s1, This article contains Supplemental Figures S1 and S2 and Tables S1–S5.
