# Supplementary figures and images for: S-Palmitoylation of Synaptic Proteins as a Novel Mechanism Underlying Sex-Dependent Differences in Neuronal Plasticity
Source: Int J Mol Sci. 2021 Jun 10;22(12):6253. doi: 10.3390/ijms22126253 (PMC8230572; doi:10.3390/ijms22126253)

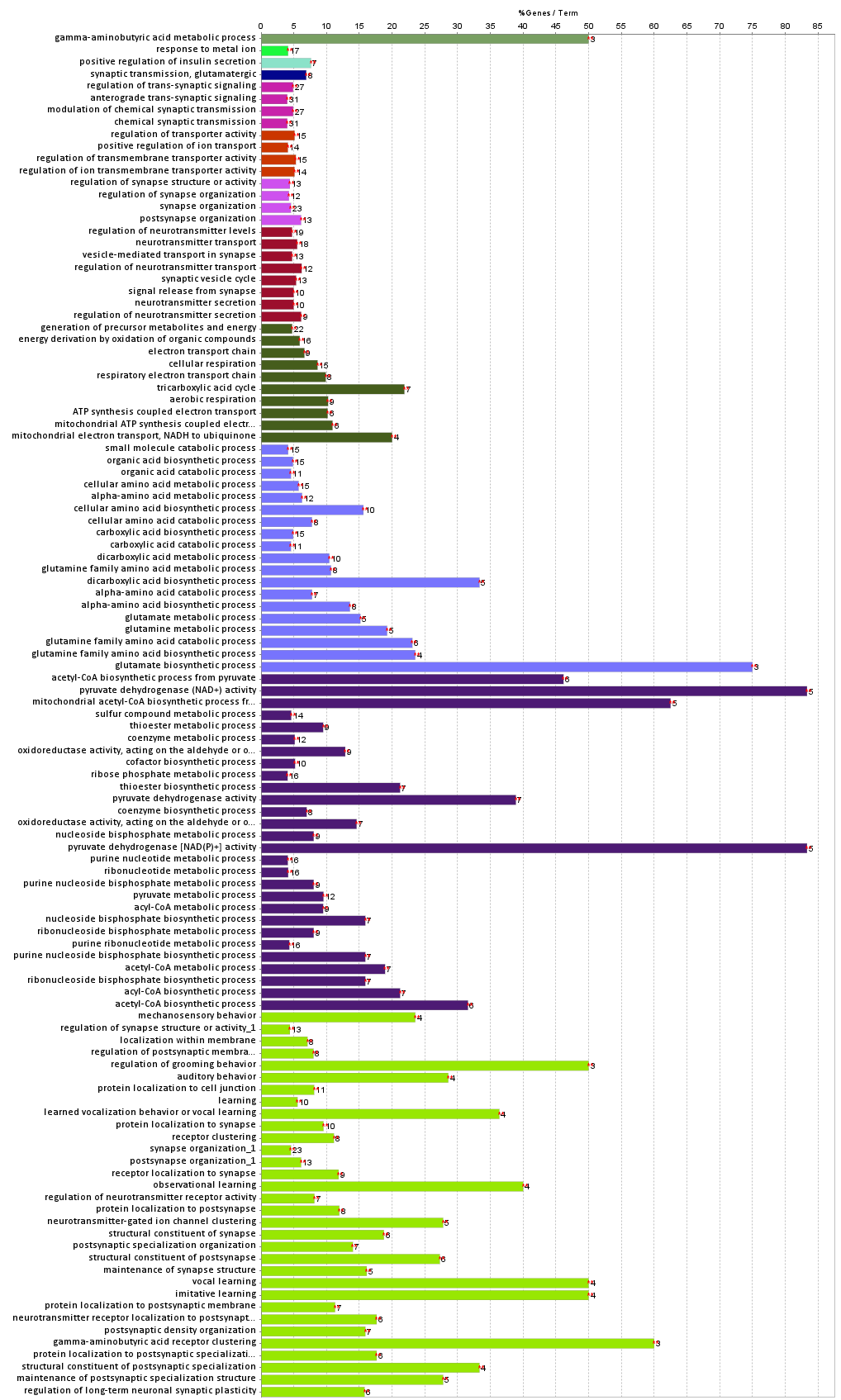

Supplement: Supplementary file 1 [file ijms-22-06253-s001.zip › Supplementary File(s)/Figure S1.png]

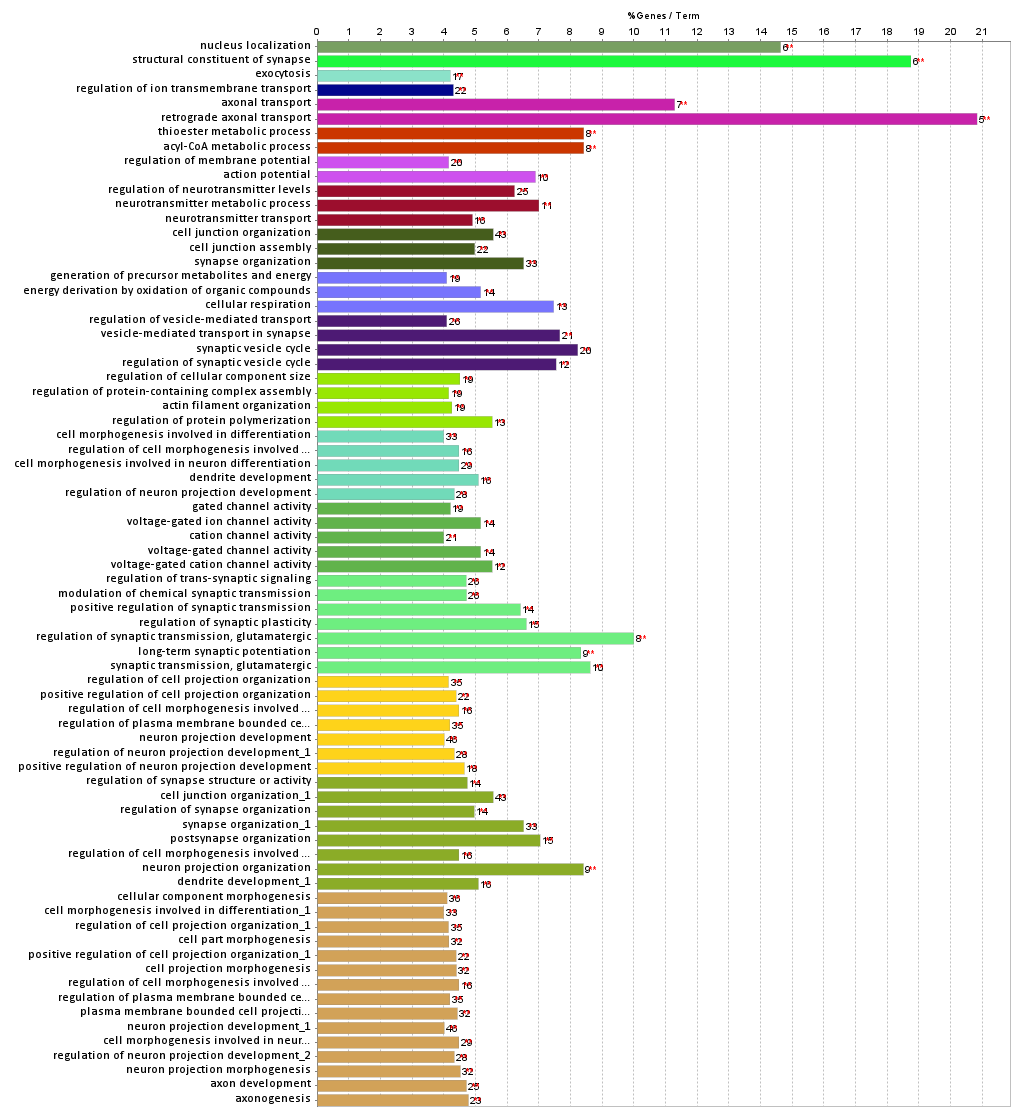

Supplement: Supplementary file 1 [file ijms-22-06253-s001.zip › Supplementary File(s)/Figure S2.png]
